# Effective Postoperative Pain Management in Thoracic Outlet Syndrome Surgery: The Role of the Erector Spinae Plane Block

**DOI:** 10.7759/cureus.48944

**Published:** 2023-11-17

**Authors:** Mariana Barros, Tania Carvalho, Ana C Pires, Gabriela Teixeira, Helder Cardoso

**Affiliations:** 1 Anesthesiology, Centro Hospitalar Tâmega e Sousa, EPE, Penafiel, PRT; 2 Vascular Surgery, Centro Hospitalar Tâmega e Sousa, EPE, Penafiel, PRT

**Keywords:** rib segment resection, first rib resection, thoracic outlet syndrome, erector spinae block, postoperative pain management

## Abstract

Thoracic outlet syndrome (TOS) often necessitates surgical intervention to alleviate neurovascular bundle compression, which can result in severe postoperative pain. The myriad of surgical techniques available for TOS treatment, the intricate involvement of diverse sensory pathways, and the limited literature on effective analgesic methods for these specific cases underscore the need for successful approaches.

This report introduces an efficacious multimodal analgesic strategy that incorporates the erector spinae plane (ESP) block to enhance postoperative pain management after a supraclavicular surgical approach. By combining this fascial block with a comprehensive rationale for its implementation, this case offers valuable insights into improving the postoperative care of TOS patients, ultimately aiming to enhance their comfort and recovery.

## Introduction

Thoracic outlet syndrome (TOS) results from the compression of the brachial plexus or the subclavian vessels at the thoracic outlet [1,2]. Surgical treatment is often the primary choice in cases of vascular compression [1], and postoperative pain can be severe, as it involves the incision of skin, fascia, muscles, and bone. However, there is limited literature on the most suitable analgesic techniques in these cases.

Our case report describes a successful multimodal analgesic technique combined with the erector spinae plane (ESP) block, employed to effectively manage postoperative pain.

## Case presentation

A 25-year-old male, 70 kg, American Society of Anesthesiologists (ASA) II with a congenital solitary kidney was diagnosed with symptomatic venous TOS accompanied by subclavian vein thrombosis. Initial treatment consisted of catheter-directed thrombolysis, which upon control venography revealed a stenosis in the costo-clavicular segment of the subclavian vein. The patient remained on anticoagulation therapy until the decompression surgery.

**Figure 1:**
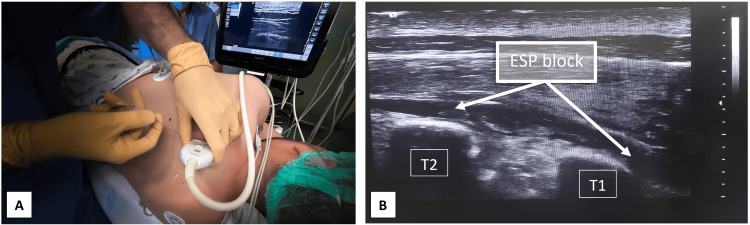
Ultrasound-guided ESP block (A) Block setup in lateral decubitus position; (B) White arrowsshowfascial plane hydro-dissected by local anesthetic ESP: erector spinae plane; T1: transverse process of first thoracic vertebra; T2: transverse process of second thoracic vertebra

After informed consent, the patient underwent partial first rib excision via a supraclavicular approach. This was performed under balanced general anesthesia combined with an ESP block. For the block, he was positioned in the right lateral decubitus position and was pre-medicated with 2 mg midazolam and 100 micrograms of fentanyl. Using an ultrasound linear probe, the ESP was performed at the left T1 level with a 22G needle using an in-plane technique. A 15 mL dose of 0.375% ropivacaine was administered, showing bidirectional longitudinal dispersion of the anesthetic both caudally and cephalically beneath the muscle and superficial to the transverse process (Figure 1).

**Figure 2:**
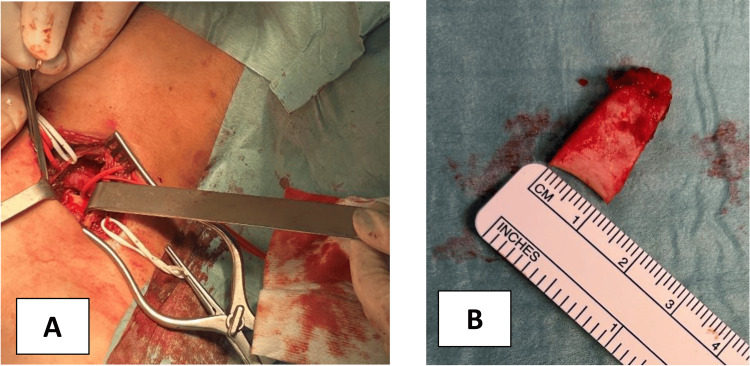
Surgical procedure (A) Supraclavicular incision; (B) Partial first rib ressection

Subsequently, general anesthesia was induced, and the removal of the first rib was executed via the supraclavicular approach, through a mid-clavicular incision, as shown in Figure 2.

Intraoperatively, analgesic medication was administered as follows: an additional 0.15 mg of fentanyl, 1 g of paracetamol, 30 mg of ketorolac, and 100 mg of tramadol. Additionally, surgical wound infiltration was performed using 5 ml of 0.75% ropivacaine.

The patient's intraoperative hemodynamics remained stable, leading to an uneventful extubation. In the post-anesthesia care unit, he reported a pain level of 1 on the numerical rating scale for pain. Pain management over the next 48 hours consisted of 1 g paracetamol IV every six hours, 30 mg ketorolac IV every six hours, and 100 mg tramadol every eight hours. He needed no morphine rescue doses. His pain scores during the initial 48 postoperative hours were 0 at rest and 2 with movement. Throughout this period, he displayed no signs of pain, discomfort, nausea, vomiting, drowsiness, or other side effects from the prescribed analgesics.

## Discussion

TOS can be categorized into three distinct conditions based on the structure under compression: (i) arterial (involving the axillary-subclavian artery), (ii) neurogenic(relating to the brachial plexus nerves), and (iii) venous (affecting the axillary-subclavian veins). Manifestations of these conditions might include limb pallor, muscle weakness during exertion of the involved limb, paresthesias, muscle atrophy, pain, and venous thrombosis [3]. Presently, four primary surgical techniques address TOS: the transaxillary, supraclavicular, infraclavicular, and posterior approaches [4]. In the transaxillary approach, the incision is made in the axilla, between the pectoralis major and latissimus dorsi muscles. In thesupraclavicular approach, the incision is positioned approximately 1-2 cm above the clavicle. This is followed by the dissection of the anterior and middle scalene muscles and the resection of the first rib. In the infraclavicular approach**, **the incision is made 1-2 cm below the clavicle, and both the subclavius muscle and the first rib are excised. In the posterior approach, this method involves an incision between the spinous processes and the medial border of the scapula.

In the present case, the supraclavicular technique was employed for the partial excision of the first rib. It is worth noting that there is limited literature on the most suitable analgesic strategy for this surgical method. The supraclavicular approach affects an anatomical region abundant in sensory pathways. Specifically, these include: (i) High cervical spinal nerves via the cervical plexus and its supraclavicular rami, (ii) Cervicothoracic spinal nerves to the brachial plexus that serve the clavicle, scalenes, and subclavian muscles, and (iii) High thoracic spinal nerves that innervate the ribs and intercostal spaces.

Considering the complex innervation of this region, selecting the right analgesic technique becomes vital when planning a regional nerve block. The literature on local-regional analgesic methods specific to this approach remains scant. One retrospective study discussed the use of the pectoral block II with the transaxillary technique, but its conclusions reported no decrease in pain scores or opioid usage [5]. Another case report highlighted the effective analgesic results of a continuous ESP block used in the transaxillary method [6].

In the planning of the current case, various analgesic strategies were considered, including high thoracic epidural block, paravertebral block, pectoserratus plane block, and serratus anterior plane block. However, thoracic epidural and paravertebral blocks are technically more challenging to perform, have notable risks, and require temporary anticoagulation suspension [7]. In contrast, the ESP block is a relatively straightforward fascial block that can be performed under anticoagulation [7]. This block involves injecting a local anesthetic into the fascial plane of the erector spinae muscle. The needle is directed towards the transverse process at the desired level, which serves as a safe stop, in this case, at the level of T1, and the local anesthetic hydro-dissects the plane beneath the erector spinae muscle. The mechanism of this block is not fully understood, but it is attributed to the possible spread to the paravertebral space and laterally to the intercostal nerves [8]. The injection into the surgical wound aims to cover the sensory nerves originating from the high cervical roots, likely not encompassed by the ESP block.

In the current case, the ESP block contributed to the required level of pain control throughout the 48-hour hospitalization period. This enhanced the patient's comfort, facilitated a swift rehabilitation process, and made an early discharge possible. Although there was no need to resort to more potent opioids, the true efficacy, safety, and potential of the ESP block in reducing opioid requirements can only be ascertained through future prospective comparative studies. 

## Conclusions

This clinical case suggests that the ESP block combined with wound infiltration can be a straightforward and valuable analgesic tool for first rib resection surgery, particularly in cases where the supraclavicular approach is used in the surgical treatment of TOS. 
